# Case report - calcification of the medial collateral ligament of the knee with simultaneous calcifying tendinitis of the rotator cuff

**DOI:** 10.1186/s12891-016-1147-z

**Published:** 2016-07-13

**Authors:** Yama Kamawal, Andre F. Steinert, Boris M. Holzapfel, Maximilian Rudert, Thomas Barthel

**Affiliations:** Department of Orthopaedic Surgery, Koenig-Ludwig-Haus, Julius-Maximilians-University Wuerzburg, Brettreichstraße 11, D- 97074 Wuerzburg, Germany

**Keywords:** Case report, Calcification, Medial collateral ligament, Knee, Rotator cuff, Open surgical repair

## Abstract

**Background:**

Calcification of the medial collateral ligament (MCL) of the knee is a very rare disease. We report on a case of a patient with a calcifying lesion within the MCL and simultaneous calcifying tendinitis of the rotator cuff in both shoulders.

**Case presentation:**

Calcification of the MCL was diagnosed both via x-ray and magnetic resonance imaging (MRI) and was successfully treated surgically. Calcifying tendinitis of the rotator cuff was successfully treated applying conservative methods.

**Conclusion:**

This is the first case report of a patient suffering from both a calcifying lesion within the medial collateral ligament and calcifying tendinitis of the rotator cuff in both shoulders. Clinical symptoms, radio-morphological characteristics and macroscopic features were very similar and therefore it can be postulated that the underlying pathophysiology is the same in both diseases. Our experience suggests that magnetic resonance imaging and x-ray are invaluable tools for the diagnosis of this inflammatory calcifying disease of the ligament, and that surgical repair provides a good outcome if conservative treatment fails. It seems that calcification of the MCL is more likely to require surgery than calcifying tendinitis of the rotator cuff. However, the exact reason for this remains unclear to date.

## Background

The medial collateral ligament is a very complex apparatus, connecting the medial surface of the femoral condyle to the tibia. Its function is to resist forces applied from the outside of the knee preventing the medial or inner part of the joint from widening. Moreover the MCL is considered a static stabilizer. Its structure is triangular and little expansible. Its origin is located proximally of the medial epicondyle of the femur next to the adductor tubercle, whereas its attachment lies below the medial condyle of the tibia on its medial surface [1].

Calcifying tendinitis is referred to as a pathological condition that is characterized by deposition of calcium-phospate particles - in their crystalline form hydroxyapatite - within a tendon. The aetiology and the factors that predispose to the development of symptoms are still not entirely clear. Different hypotheses have been proposed in the literature but most of them are still not proven or under investigation [2, 3] Some authors report that calcification is associated with other pathologic conditions such as renal failure, collagen vascular disease (e.g. dermatomyositis or scleroderma), neurological disorders, Vitamin D overload, tumoral calcinosis or dystrophic calcification [4].

The pathophysiological cascade involves a locally decreased oxygen tension within the affected tissue that leads to fibrocartilaginous metaplasia and eventually to calcification of the fibrocartilaginous matrix [4]. This condition is typically associated with a morphologically intact outer structure, in our case an intact rotator cuff and MCL, with a negative history of any trauma. Four stages of calcifying tendinitis have been described: a precalcific, calcific, resorptive, and postcalcific stadium [5].

The typical first clinical manifestation is subacute low-grade pain that commonly increases during nighttime. Later, during the resorptive stage which involves vascular invasion and migration of phagocytic cells and the development of an edema leading to increased intratendinous pressure, the disease is characterized by sharp acute pain limiting the range of motion of the affected joint [4].

As calcifying tendinitis is usually a self-limiting condition, conservative treatment should be the first line therapy [6–8].

Calcification of the MCL is a very rare disease. In the recent literature only three reports on single symptomatic calcifications of the medial collateral ligament of the knee joint can be found. None of these reports describes the involvement of other parts of the body. To our knowledge, we are the first to demonstrate a case of a patient suffering from both a calcification of the medial collateral ligament of the knee and calcifying tendinitis of the rotator cuff [9–11].

## Case presentation

A 50-year-old caucasian woman was referred to our clinic with a 12 months history of severe, recurrent pain episodes in her right knee. She was a non-smoker, with no other notable family or medical history for any pathological condition. Systemic diseases such as gout, systemic sclerosis, dermatomyositis, and sarcoidosis or any metabolic or endocrine disorders such as hyperparathyroidism and renal failure were excluded. There were no past trauma incidents. Her orthopedic surgeon had diagnosed a calcifying lesion of the MCL 9 months prior to admission to our hospital and had treated her conservatively under the assumption of a calcifying tendinitis-like pathological condition. After some temporary pain relief she still suffered from pain at the medial side of the knee and limited range of motion. The conservative management included analgesics, non-steroidal anti-inflammatory drugs (NSAIDs), electro-therapy and shock wave therapy.

**Fig. 1:**
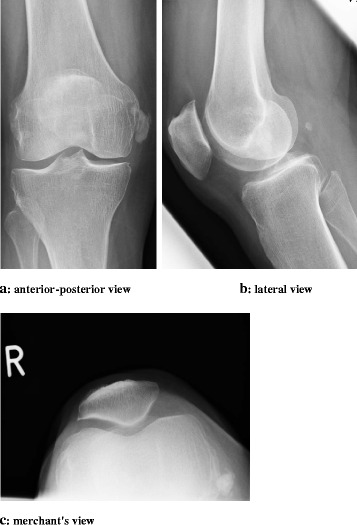
Calcification body within the proximal and middle section of the MCL. It appears as homogeneous and round to ovoid calcification in the soft tissue with well defined margins

**Fig. 2:**
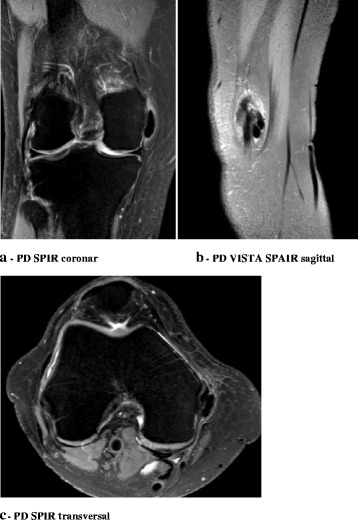
Rounded area of low signal intensity on all imaging sequences in direct vicinity of the intact MCL, laying directly on the ligament structure

Physical examination of the right knee revealed local pain and swelling over the medial femoral condyle with intact soft tissues and no joint effusion. The patient was able to fully extend her knee but flexion was limited to 120° with increasing pain at the MCL beginning with 90° flexion. Patellar mobility was not limited. There was no ligament laxity and clinical tests for detection of meniscus lesions were all negative. There was no sensorimotor deficit. Blood tests showed no evidence of haematological or metabolic abnormality. Knee x-rays revealed linear soft tissue opacity medial to the femoral condyle, suggesting a lobate calcifying lesion within the proximal and middle section of the MCL and surrounding tissue. Antero-posterior, lateral and merchant’s views verified the presence of the calcified lesion. MRI without contrast agent of the knee confirmed the presence of this lobate structure, which was characterized by an hypointense signal on the T2 weighted images, located in direct vicinity of the intact MCL in the coronal and sagittal plane, with direct contact to the ligament structure, which was seen in the sagittal and axial plane images of the knee. There were no signs for a mature bony lesion (Figs. 1 and 2).

Due to failure of conservative treatment regimes, we recommended surgery and performed an arthroscopy followed by an open procedure with removal of the calcified lesion. Intraoperatively, we found areas of chondromalacia grade 2 of the patellofemoral compartment and the medial femur condyle. The calcification body was directly attached to the MCL, which was structurally intact. Its consistency was toothpaste-like analogous to the macroscopic appearance of a calcifying tendinitis of the shoulder. The directly adhering parts of the soft tissues were also removed.

There were no complications during the postoperative course. Weight bearing started with 20 kg and increased to full weight bearing over 2 weeks, which was well tolerated.

**Fig. 3:**
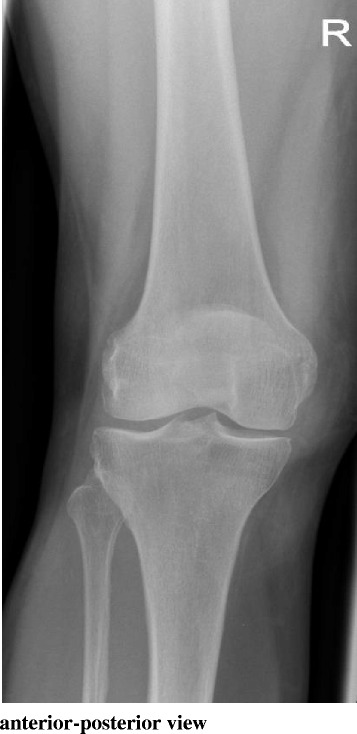
X-ray picture 4 months after surgery. Calcification deposit completely removed

After wound healing and recovery of the soft tissues, the patient was pain-free after 4 weeks. Clinical examination and radiographic evaluation in the next 12 months demonstrated no signs of recurrence without any clinical symptoms (Fig. 3).

During these follow up examinations, the patient described recurrent symptoms of pain and restricted movement of the right shoulder, which had persisted previously for over a year. She had suffered from calcifying tendinitis in both of her shoulder joints 17 years ago. Back then, the x-ray pictures had shown calcification bodies near to the greater tubercle at the insertion of the supraspinatus tendon. These lesions had been treated successfully using shockwaves at that time, and had not re-appeared since.

A current MRI survey demonstrated a rupture of the supraspinatus tendon. The patient successfully underwent a shoulder arthroscopy with a rotator cuff repair in our department. Surgery revealed signs of degenerative changes of the tendon, which had led to the rupture, but no evidence for residual calcium deposits within the supraspinatus tendon were found (Table 1).

**Table 1:** Timetable

Dates	Relevant Past Medical History and Interventions		
	50-year-old caucasian woman, non-smoker, with no other notable family or medical history for any pathological condition. Systemic diseases such as gout, systemic sclerosis, dermatomyositis, and sarcoidosis or any metabolic or endocrine disorders. There were no past trauma incidents.		
25.11.1997	She had suffered from calcifying tendinitis in both of her shoulder joints 17 years ago. Back then, the x-ray pictures had shown calcification bodies near to the greater tubercle. These lesions had been treated successfully using shockwaves at that time, and had not reappeared since then.		
Dates	Summaries from Initial and Follow-up Visits	Diagnostic Testing (including dates)	Interventions
Since September 2013	history of severe, recurrent pain episodes in her right knee		
10.12.2013	a calcifying lesion of the MCL was diagnosed	x-rays, ultrasound, MRI	conservative management included analgesics, non-steroidal anti-inflammatory drugs, electro-therapy and shock wave therapy
18.09.2014	Due to failure of conservative treatment regimes, we recommended surgery		arthroscopy followed by an open procedure with removal of the calcified lesion.
04.03.2015	rupture of the supraspinatus tendon.	MRI	shoulder arthroscopy with a rotator cuff repair
31.10.2014 08.01.2015 09.04.2015 02.10.2015	Clinical examination and radiographic evaluation demonstrated no signs of recurrence without any clinical symptoms	x-rays	

## Discussion and conclusions

Articular calcification deposits are most commonly found within the shoulder. Calcifying tendinitis, is one of the most frequent etiologies for shoulder pain. The supraspinatus tendon (80 % of cases), followed by the infraspinatus (15 % of cases) and subscapularis (5 % of cases) tendon, is most commonly affected [8]. The prevalence for these lesions is 3–20 % in asymptomatic patients and 7–54 % in symptomatic patients, reaching its peak between 30–50 years of age [2]. Women seem to be affected more often than men, at a ratio of 3 to 2 [7].

After the shoulder, the hip is the second frequent location for the development of calcification deposits, most commonly found in the gluteus medius tendon at the greater trochanter. Furthermore the posterolateral femoral attachment of the gluteus maximus can be involved. Other areas of involvement include the iliopsoas tendon insertion at the lesser trochanter, and the ischial origins of the common hamstring tendons [12].

Another joint which can be affected is the elbow, including the bursa and the flexor and extensor tendon complexes at the epicondylar origins, the triceps, brachialis, and biceps tendons. It can also occur in the medial and lateral collateral ligamentous complexes [13, 14].

Other parts of the upper extremity that can be involved are the wrist and the hand. The wrist is more frequently affected than the hand. The pisiform insertion site of the flexor carpi ulnaris tendon is reportedly the main site of involvement in the wrist. In the hand, deposits in the metacarpophalangeal and interphalangeal regions are not uncommon [15].

In the ankle and foot, calcifications can involve a plethora of structures, among them the flexor hallucis longus and brevis and the peroneus tendons [16].

In the knee, calcification deposits can occur in the joint, on the extrasynovial anterior or posterior cruciate ligament [17, 18]. Usually they can be found near osseous attachments of the ligaments or the popliteus tendon rather than at other structures [3, 19, 20].

Calcification deposits typically present on MR imaging as rounded areas of low signal intensity on all imaging sequences. The deposits are particularly conspicuous on gradient echo imaging. In the acute symptomatic phase the process has an aggressive appearance with marrow and soft tissue edema that may mimic infection, trauma, or neoplasm [21].

An association between acute pain attacks and histological evidence of calcium resorption has been described previously [7]. Most symptoms resolve within 2–3 weeks under conservative treatment. NSAIDs are an essential part of the basic treatment strategy. If there is no relief of the symptoms under NSAIDs or the drugs cannot be tolerated, local corticosteroid injections, oral steroids, shockwave therapy and needling are further therapeutic options. If conservative management fails, deposits can be removed surgically or via image-guided aspiration [22].

Posttraumatic calcification can appear in different locations, including the medial collateral ligament. The term ‘Pellegrini-Stieda lesion’ is used for the latter finding, and is named after both doctors Pellegrini and Stieda, who described this phenomenon for the first time in 1905 and 1907, respectively. However, it was suggested by Koenig, Koehler and Pfister in 1909, that there are Pellegrini-Stieda shades, who aren’t necessarily caused by trauma and may have other etiologies [23–25].

The Pellegrini-Stieda disease is a relatively rare phenomenon and is commonly associated with sports injuries. It is thought that Pellegrini-Stieda lesions are post-traumatic ossifications following an avulsion injury to the attachment of the medial collateral ligament, at the medial femoral condyle. Tearing fibres of the ligament at its superior femoral attachment can cause hematoma or inflammatory edema. The soft tissue can be also affected and absorbs calcium salts during the later stages of the disease. This mechanism takes place approximately 2–6 weeks after the trauma. In the next stage of the disease, the calcium salts can be resorbed which results in a degradation of the lesion or an ossified mesh can develop, which usually gets connected to the femoral condyle by a pedicle within the next 6 months. The calcification of the superior femoral attachment is in most cases characteristic, confirmed by x-ray and often associated with ruptures of the anterior cruciate ligament [26, 27].

Calcification deposits typically present on MRI imaging as rounded areas of low signal intensity on all imaging sequences. The deposits are particularly conspicuous on gradient echo imaging. In the acute symptomatic phase the process has an aggressive appearance with marrow and soft tissue edema that may mimic infection, trauma, or neoplasm [21].

Most of the patients with this post-traumatic calcification of the MCL are asymptomatic. The term Pellegrini-Stieda syndrome is only used if the symptoms can be directly associated with the appearance of the Pellegrini-Stieda shadow [28]. They can increase within a few weeks or months, and can result in nearly completely restricted range of motion. The pain and swelling are located on the medial side of the knee [29–31].

The kind of calcification of the MCL presented in our case seems to be similar to the Pellegrini-Stieda syndrome at the first glance, but has to be distinguished from this entity in terms of its pathophysiology and -morphology. The calcification body in our case was not localized at the insertion of the ligament and also differed in its radiological morphology and appearance. Ossification of Pellegrini-Stieda type lesions are of concave and flattened, whereas the calcification seen in our case was rounded and lobate, clearly circumscribed and dense, corresponding to type 1 of the radiological classification of calcifying tendinitis by Gaertner and Heyer [32]. The intraoperative paste-like findings corresponded to the intraoperative characteristic appearance of a calcifying tendinitis of the rotator cuff. Therapy was analogous to the management of this well known pathologic entity of the rotator cuff. After all conservative treatment modalities were exhausted, surgical excision was performed. The postal-surgical course was uneventful. Six months after the surgery, the patient was completely free of pain and was able to perform her everyday activities without any limitation.

This is the first reported case of a patient suffering from both a calcifying lesion of the medial collateral ligament of the knee and calcifying tendinitis of the rotator cuff in both shoulders.

In the literature three reports with seven cases describing single symptomatic calcifications of the medial collateral ligament of the knee can be found. Five patients were successfully treated by surgical resection, two patients were treated conservatively with a positive result. There were no reports that one of them had other parts of the body involved in a calcification process [9–11].

In the precedent medical history of our patient, she had suffered from calcifying tendinitis in both shoulders, and had successfully been treated conservatively. Because of a rupture of the tendon of the musculus supraspinatus the patient required surgery 17 years later, which revealed no remaining or recurrent calcification bodies.

Our experience suggests that magnetic resonance imaging and x-ray evaluation are invaluable tools in the diagnosis of this condition and that surgical repair provides a good outcome, if conservative treatment fails.

In contrast to calcifying tendinitis of the rotator cuff, calcification of the MCL is a very rare disease. Because the patient in our report suffered from both of these diseases, one can come to the conclusion that there is probably the same kind of etiology, however, the exact mechanism of the calcium deposition involved in these two conditions is not entirely clear. Analogous to the rotator cuff, conservative treatment regimes can be used, although it seems that the calcification of the MCL is a condition that is more resistant to conservative therapy than common calcifying tendinitis of the rotator cuff. A possible reason could be the difference in the pathomechanism of the two lesions. It is well known that the integrity of the rotator cuff can be affected due to pathological mechanisms such as inner and outer impingement leading to structural changes. On the other hand the pathological mechanisms leading to an impairment of the MCL are different. In general, the MCL is damaged by extrinsic indirect stresses which might result in different structural changes as seen in the tendon [33, 34].

## Abbreviations

MCL, medial collateral ligament; MRI, magnetic resonance imaging; NSAIDs, nonsteroidal anti-inflammatory drugs
